# The Effect of Dietary Protein Concentration on the Fecal Microbiome and Serum Concentrations of Gut-Derived Uremic Toxins in Healthy Adult Cats

**DOI:** 10.3390/vetsci10080497

**Published:** 2023-08-02

**Authors:** Stacie Summers, Jessica Quimby, Jason Gagné, Michael Lappin

**Affiliations:** 1 Carlson College of Veterinary Medicine, Oregon State University, Corvallis, OR 97331, USA; 2 College of Veterinary Medicine, The Ohio State University, Columbus, OH 43210, USA; quimby.19@osu.edu; 3 Nestlé Purina Pet Care Company, St. Louis, MO 63102, USA; jason.gagne@purina.nestle.com; 4 College of Veterinary Medicine, Colorado State University, Fort Collins, CO 80523, USA; michael.lappin@colostate.edu

**Keywords:** uremic toxin, indoxyl sulfate, p-cresol sulfate, microbiome, metagenomics

## Abstract

**Simple Summary:**

Dietary formulation can affect the composition of the microbes that reside in the gut and microbial products. Some microbial products are toxins that can accumulate in the systemic circulation of cats with a disturbed gut microbial community. Indoxyl sulfate, p-cresol sulfate, and trimethylamine-n-oxide are toxins produced during microbial fermentation of protein in the gut. By changing the gut microbial community, dietary protein concentration could affect the production of these toxins. The purpose of this study was to evaluate the effect of feeding healthy adult cats with foods containing variable protein concentrations for 3 months on the fecal microbiome and serum concentrations of the major gut-derived toxins. We found that cats fed a high-protein diet had increasing serum concentrations of p-cresol sulfate in the first 8 weeks before returning to baseline concentrations. Cats fed a high-protein diet also had increased diversity of the fecal microbial community and reduced relative abundance of beneficial bacteria in the *Bifidobacterium* genus.

**Abstract:**

The purpose of this study was to evaluate the effect of feeding healthy adult cats with foods containing variable protein concentrations on the fecal microbiome and serum concentrations of the gut-derived uremic toxins indoxyl sulfate, p-cresol sulfate (pCS), and trimethylamine-n-oxide. Twenty healthy young adult cats were randomized into two groups and fed either a low-protein diet (LPD; 7.4 g/100 kcal ME) or a high-protein diet (HPD; 11.0 g/100 kcal ME) for a 12-week period. Serum uremic toxin concentrations were measured via liquid chromatography tandem mass spectrometry, and the fecal microbiome was characterized using shallow sequence shotgun metagenomics. Cats that consumed the HPD had higher pCS concentrations at 8 weeks (*p* = 0.028) when compared to baseline. After 12 weeks, cats fed the HPD had higher fecal alpha diversity indices at both the taxonomic and functional levels and lower fecal *Bifidobacterium* relative abundance compared to those cats fed the LPD. In conclusion, a change in diet and dietary protein concentration shifted the fecal microbial community and microbial function. Feeding cats a high amount of protein increased serum concentrations of the uremic toxin pCS; however, the effect was short-lived.

## 1. Introduction

The intestinal microbiome is important for the maintenance of host health and prevention of disease. Dietary nutrients play a major role in the intestinal microbial composition and function [1]. Intestinal microbes can ferment substrates from the diet, such as protein, and alter the microbiome and metabolome in healthy people, dogs, and cats [2,3,4,5]. Fermentation of protein by proteolytic bacteria produces bacterial-derived metabolites, a process called putrefaction, and these postbiotic metabolites directly affect host health. Indoxyl sulfate (IS), p-cresol sulfate (pCS), and trimethylamine-n-oxide (TMAO) are gut-derived uremic toxins produced from endogenous and colonic fermentation of amino acids by microbiota. These metabolites contribute to morbidity and mortality in people with kidney disease and have been shown to accumulate in cats with chronic kidney disease (CKD) [6,7]. This accumulation is related to reduced renal elimination and enhanced generation of these toxins by a disturbed gut microbiome [8,9,10].

In people, dietary management strategies can successfully manipulate production of these gut-derived uremic toxins. Low protein intake in people can reduce uremic toxin generation by the gut microbiota and thus the accumulation of these toxins in the systemic circulation. In those with kidney disease, low protein intake can be used to manage signs of uremia and delay progression of disease [11,12,13,14]. The benefits of feeding cats a renal therapeutic diet to restrict protein and phosphorus intake have shown similar benefits [15,16,17]. These cat foods are often similar to or below the minimum protein concentration of 26% on a dry matter (DM) basis (or 6.5 g/100 kcal) recommended by the Association of American Feed Control Officials (AAFCO) Cat Food Nutrient Profile for adult maintenance [18]. Limited information is available on cats regarding the effect of protein intake on uremic toxin accumulation. One study in cats with International Renal Interest Society (IRIS) stage 1 CKD showed higher plasma IS and pCS concentrations when cats were fed a high-protein diet (8.0 g/100 kcal ME) compared to a diet with a lower concentration of protein (5.7 g/100 kcal ME) [19]. To our knowledge, there are no reports evaluating the correlation between the fecal microbiome and serum concentrations of gut-derived uremic toxins in cats fed diets with variable protein concentrations.

We hypothesized that diets with variable protein concentrations would differentially alter the gut microbiome of healthy cats and correlate to changes in serum gut-derived uremic toxin concentrations. We also hypothesized that a high-protein diet would increase serum concentrations of gut-derived uremic toxins. The objective of the study was to evaluate the effect of feeding cats diets with variable protein concentrations (7.4 g/100 kcal or 11.0 g/100 kcal) on the fecal microbiome and serum concentrations of the major gut-derived uremic toxins IS, pCS, and TMAO in healthy adult cats.

## 2. Materials and Methods

### 2.1. The Study Design and Cats

This study was a prospective randomized parallel diet trial using healthy cats (*n* = 20), and the protocol followed the American Association for Accreditation of Laboratory Animal Care guidelines. Cats were of domestic shorthair breed, 3 years of age, and of mixed sex (10 castrated males (CM); 3 spayed females (FS); 7 intact females (FI)). Health status was determined via a physical examination (including body condition score (BCS) and muscle condition score (MCS)) and minimum database including complete blood count (CBC), serum biochemistry panel, urinalysis, and serum total thyroxine concentration. At enrollment, all cats had a normal physical examination, apart from one female cat with a grade II/VI left parasternal heart murmur of unknown etiology. All cats had a normal minimum database, serum thyroxine concentrations, and urinalysis including serum creatinine concentration < 1.8 mg/dL and urine specific gravity (USG) > 1.030.

Cats were randomized into 2 groups of 10 cats blocked by sex using an online randomizer tool (low-protein diet (LPD) group: 5 CM, 2 FS, 3 FI; high-protein diet (HPD) group: 5 CM, 1 FS, 4 FI). Cats were housed in their communal groups and separated by room for the study duration. The cats had access to perches and toys in their rooms and socialized daily. After acclimation to rooms for a week, cats were transitioned to study diets and fed ad libitum for 3 months. The communal rooms and litter boxes were cleaned daily, and cats had access to fresh food and water ad libitum. The criterion for removal of a cat from the study included signs of illness (i.e., vomiting, diarrhea), refusal to eat the study diet, or weight loss greater than 10% of body weight.

Blood, urine, voided feces were collected at baseline (Week 0) and every 4 weeks during the 3-month study period (Weeks 4, 8, 12). Physical examinations, body weights, and additional assays, including a CBC, serum biochemistry panel, and urinalysis, were performed at each sample collection to reevaluate health status. For fecal collection, the cats were separated using wire kennels with a solid floor, perch, litterbox, water bowl, and food bowl for 36–48 h. The fecal samples were aliquoted and transferred to a −80 °C freezer within 12 h of defecation. A fecal score (1–7-point scale; Nestlé Purina, St. Louis, MO, USA) was obtained for each cat. After an overnight 12 h fast, each cat was sedated with an intravenous injection of ketamine (20 mg/cat) and midazolam (5 mg/cat). Body weight was obtained, blood was collected via jugular venipuncture, and urine was collected via cystocentesis.

Sample collection and processing were standardized to minimize pre-analytical variation, including the cat order for blood sampling and use of a single operator. Blood was centrifuged for 10 min. Serum and urine samples were aliquoted and then stored at 4 °C for less than 3 h before being transferred to a −80 °C freezer.

### 2.2. Foods

The pre-trial diet, LPD, and HPD were only available in a dry form. Cats were fed the pre-trial diet ad libitum for at least 3 months prior to the study. The pre-trial diet was an over-the-counter adult dry cat food (Meow Mix Original Choice, Big Heart Pet Inc., San Francisco, CA, USA) and was formulated to meet the AAFCO Cat Nutrient Profiles for all life stages according to the nutritional adequacy statement on the food label. The pre-trial diet and study diets were analyzed for macronutrient composition and fiber concentrations by Nestlé Purina Analytical Laboratories (St. Louis, MO, USA). The diets were homogenized before analysis. Methods validated by the Association of Official Agricultural Chemists International were used to measure dietary amino acid concentrations, moisture, crude protein, crude fiber, total dietary fiber (soluble and insoluble), crude fat by acid hydrolysis, and ash. The carbohydrate content of the diets was calculated by difference (100 minus crude protein, crude fiber, crude fat, ash, and moisture). Mineral concentrations were measured via inductively coupled argon plasma emission spectroscopy.

Food composition, including the amino acid concentrations, of the pre-trial diet, LPD, and HPD can be found in Table 1. The pre-trial diet contained 8.7 g/100 kcal of protein. Study diets were formulated and prepared by a food manufacturer (Nestlé Purina, St. Louis, MO, USA) specifically for the diet trial. The study diets were formulated with the same ingredients and differed primarily in protein concentration (LPD, 7.4 g/100 kcal; HPD, 11.0 g/100 kcal). The LPD was approximately 0.5% and 13.5% higher in fat and carbohydrates, respectively, than the HPD. Both diets had a phosphorus concentration (LPD, 107 mg/100 kcal; HPD, 117 mg/100 kcal) below the AAFCO Cat Food Nutrient Profile established minimum for maintenance of healthy adult cats (125 mg/100 kcal) [18].

### 2.3. Serum Uremic Toxin Assay

Serum total IS, pCS, and TMAO concentrations were measured via liquid chromatography with tandem mass spectrometry, as previously described [20].

### 2.4. Shallow Shotgun Metagenomic Sequencing

Voided fecal samples obtained at Week 0 and Week 12 were shipped frozen on dry ice to an outside laboratory for shallow shotgun metagenomic sequencing of stool DNA (BoosterShot, Diversigen, Houston, TX, USA). Nucleic acids were extracted from the stool samples with a QIAmp PowerFecal DNA kit (Qiagen, Venlo, Netherlands) automated for high throughput on QiaCube (Qiagen), with bead beating in 0.1 mm glass bead plates. Libraries were prepared with a procedure adapted from the Nextera Library Prep kit (Illumina, San Diego, CA, USA). Libraries were sequenced on an Illumina NextSeq using single-end 1 × 150 reads with a NextSeq 500/550 High Output v2 Kit (Illumina). DNA sequences were filtered for low quality (Q-Score < 30) and length (<50), and adapter sequences were trimmed using cutadapt. Fastq files were converted into a single fasta using shi7. Sequences were trimmed to a maximum length of 100 bp prior to alignment.

DNA sequences were aligned to a curated database containing all representative genomes in RefSeq for bacteria with additional manually curated strains [21]. Alignments were made at 97% identity against all reference genomes. Every input sequence was compared to every reference sequence in the Diversigen Venti database using fully gapped alignment with BURST [22]. Ties were broken by minimizing the overall number of unique operational taxonomic units (OTUs). For taxonomy assignment, each input sequence was assigned the lowest common ancestor that was consistent across at least 80% of all reference sequences tied for best hit. OTUs accounting for less than one-millionth of all species-level markers and those with less than 0.01% of their unique genome regions covered (and <1% of the whole genome) were discarded. The number of counts for each OTU was normalized to the average genome length. Samples with fewer than 10,000 sequences were also discarded. The count data were then converted to relative abundance for each sample. The normalized and filtered tables were used for all downstream analyses.

Kyoto Encyclopedia of Genes and Genomes Orthology groups (KEGG KOs) were observed directly using alignment at 97% identity against a gene database derived from the strain database used above [23]. To construct this database, a representative strain for each species in the Venti database was annotated using Prokka (v 1.12) [24]. Prokka annotations were cross-referenced to KEGG identifications, and gene sequences with KEGG annotations were retained for use in the functional database.

Within-sample diversity was assessed via calculation of alpha diversity metrics (Observed OTUs, Shannon, Chao1), and between-sample differences were assessed via beta diversity measurements as Bray–Curtis dissimilarities (distances) using the OTU and KEGG Enzyme tables rarefied to the sample with the lowest sequencing depth using QIIME 1.9.1 [25].

### 2.5. Statistical Analysis

Descriptive data are presented as medians (range). Statistical analysis was performed using GraphPad Prism (GraphPad Software, La Jolla, CA, USA). Data were (natural) log-transformed to meet the assumption of normality. Residual diagnostic plots were used to evaluate model assumptions of normality and equal variance. If normality was not met, then a non-parametric test was performed on the raw values. Significance defined as *p* < 0.05.

Body weight and serum concentrations of the selected biochemical parameters were compared between Week 0 and Week 12 using a paired two-tailed Student t-test. Serum uremic toxin concentrations at baseline were compared to those taken at Weeks 4, 8, and 12 within each group using a repeated measured one-way ANOVA, with Geisser–Greenhouse correction and Holm–Sidak’s multiple comparisons tests.

For shotgun metagenomic data, differences in alpha diversity metrics (Observed OTU richness, Chao1, and Shannon) were compared between Week 0 and Week 12 within each group and between groups at each timepoint using Wilcoxon signed-rank (paired) tests and Wilcoxon rank sum (unpaired), respectively. For beta diversity analysis, Bray–Curtis dissimilarities distances were calculated using the OTU and KEGG Enzyme tables rarefied to the sample with the lowest sequencing depth. To account for repeated sampling within individual animals, differences in beta diversity between diets were assessed using GLMM-MiRKAT. Taxa (Phylum, Family, Genus, and OTU-level) and functional (KEGG L2 Pathways, L3 Pathways and Enzymes) differential abundances were assessed using the ALDEx2 R package. Count tables were transformed using the centered log-ratio (CLR) transformation, and differential abundance testing was performed with Wilcoxon tests with FDR correction for multiple hypothesis testing. Potential associations between taxa and the serum uremic toxin concentrations (IS, pCS, and TMAO) were assessed for each cat. This correlation analysis was focused on taxa that were differentially abundant between the LPD and HPD groups at Week 12. The correlations were tested between CLR-transformed abundances of significant taxa and paired uremic concentrations via Spearman’s rank correlation tests. Multiple hypothesis testing p-value corrections were carried out via the FDR method.

## 3. Results

All cats completed the study and consumed the study diets without adverse effects. Table 2 shows select physical examination parameters, fecal scores, and select biochemistry analyte concentrations at Week 0 and Week 12 for cats in both study groups. Body weight, BCS, and MCS remained static throughout the study, and no cat lost or gained >10% of body weight. Fecal score, serum BUN, creatinine, calcium concentrations, and USG were not statistically different at Week 12 compared to Week 0. Serum albumin was higher at Week 12 compared to Week 0 for both the HPD group (*p* = 0.004) and the LPD (*p* = 0.002) group. Serum phosphorus was higher at Week 12 compared to Week 0 in the HPD group (*p* < 0.001).

### 3.1. Serum Uremic Toxin Concentrations

**Figure 1:**
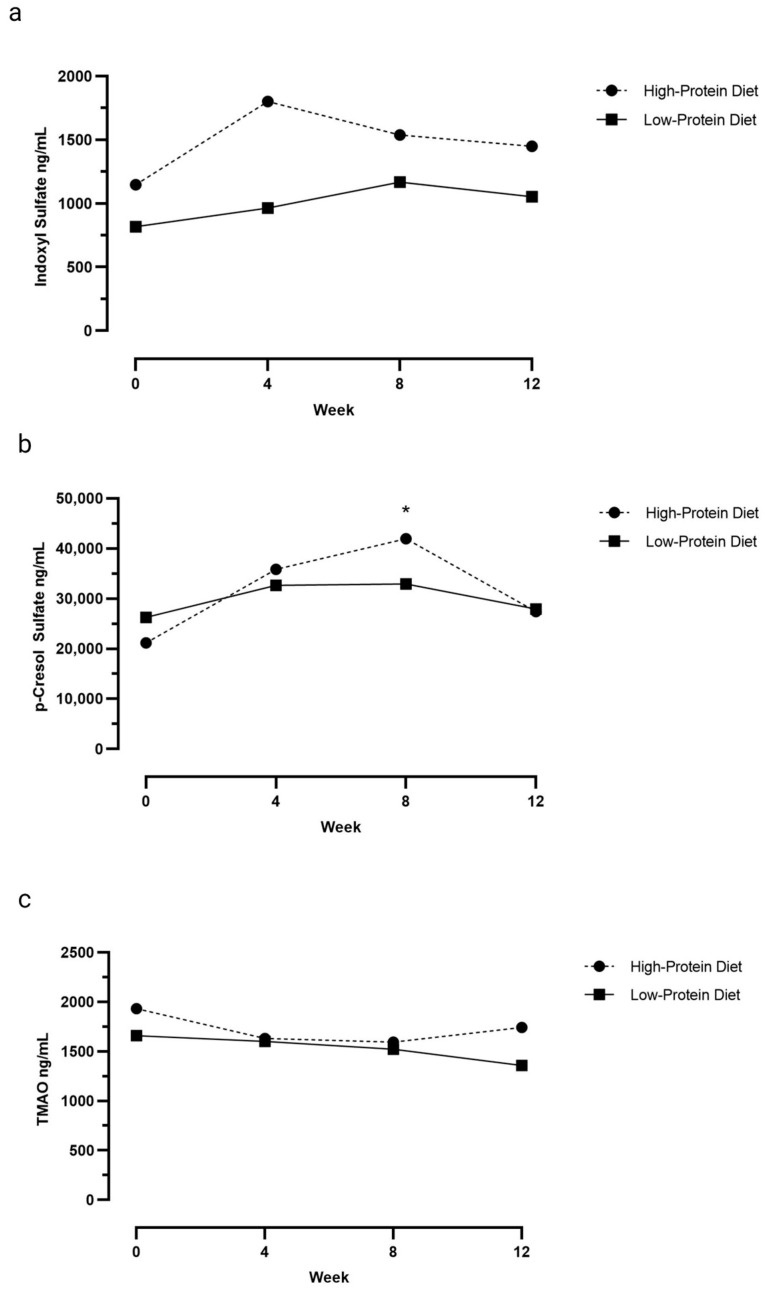
Serum indoxyl sulfate (**a**), p-cresol sulfate (**b**), and trimethyalamine-n-oxide (TMAO; (**c**)) concentrations (ng/mL) in cats at baseline (Week 0) and after being fed either a low-protein diet (*n* = 10 cats) or high-protein diet (*n* = 10 cats) for 4, 8, and 12 weeks. Line graph represents the median. Asterisks signify a statistically significant difference in serum p-cresol sulfate concentrations at Week 8 (*p* = 0.028) compared to Week 0 in cats fed the HPD.

Table 3 and Figure 1a–c show serum IS, pCS, and TMAO concentrations at Weeks 0, 4, 8, and 12 for cats fed the HPD and cats fed the LPD. In the cats fed the HPD, serum pCS concentrations increased in the first 8 weeks and were higher at Week 8 when compared to baseline (*p* = 0.028). However, serum pCS concentrations did not differ between baseline and Week 4 (*p* = 0.07) and Week 12 (*p* = 1.0). In contrast, in the cats fed the LPD, serum pCS concentrations at Weeks 4, 8, and 12 did not differ from baseline (*p* = 0.05). Serum IS (LPD: *p* = 0.5; HPD: *p* = 0.2) and TMAO (LPD: *p* = 0.2; HPD: *p* = 0.1) concentrations were not different at Weeks 4, 8, and 12 when compared to baseline values in either group.

### 3.2. Fecal Microbiome

Forty fecal samples were sequenced. The median sequencing count was 3,391,201 (range, 1,204,659–4,440,918).

#### 3.2.1. Alpha Diversity Metrics

Table 4 shows the alpha diversity metrics (Observed OTU richness, Chao1, and Shannon) at the taxonomic and functional level for the LPD and HPD groups at Week 0 and Week 12. At the taxonomic level, no significant differences in alpha diversity metrics were observed between the HPD and LPD groups at Week 0; however, the number of Observed OTUs (*p* = 0.045) and Shannon diversity (*p* = 0.019) were higher in the HPD group compared to the LPD group at Week 12. When comparing Week 0 to Week 12 samples within each group, no differences in alpha diversity were observed between the two timepoints for the LPD group; however, the Shannon diversity metric was higher at Week 12 compared to Week 0 for the HPD group (*p* = 0.10).

Similarly, at the functional level (KEGG enzymes), no significant differences in alpha diversity metrics were observed between the HPD and LPD groups at Week 0; however, significant differences between groups were observed for all three alpha diversity metrics at Week 12. The HPD group had higher Observed OTUs (*p* = 0.013), Chao1 (*p* = 0.063), and Shannon diversity (*p* = 0.029) compared to the LPD group at Week 12. When comparing the Week 0 to Week 12 samples within each group, the Observed OTU richness and Shannon diversity index were higher at Week 12 compared to Week 0 within the HPD group (*p* = 0.010 and *p* = 0.020, respectively). No significant differences were found between the two timepoints for the LPD group.

#### 3.2.2. Beta Diversity Metric

**Figure 2:**
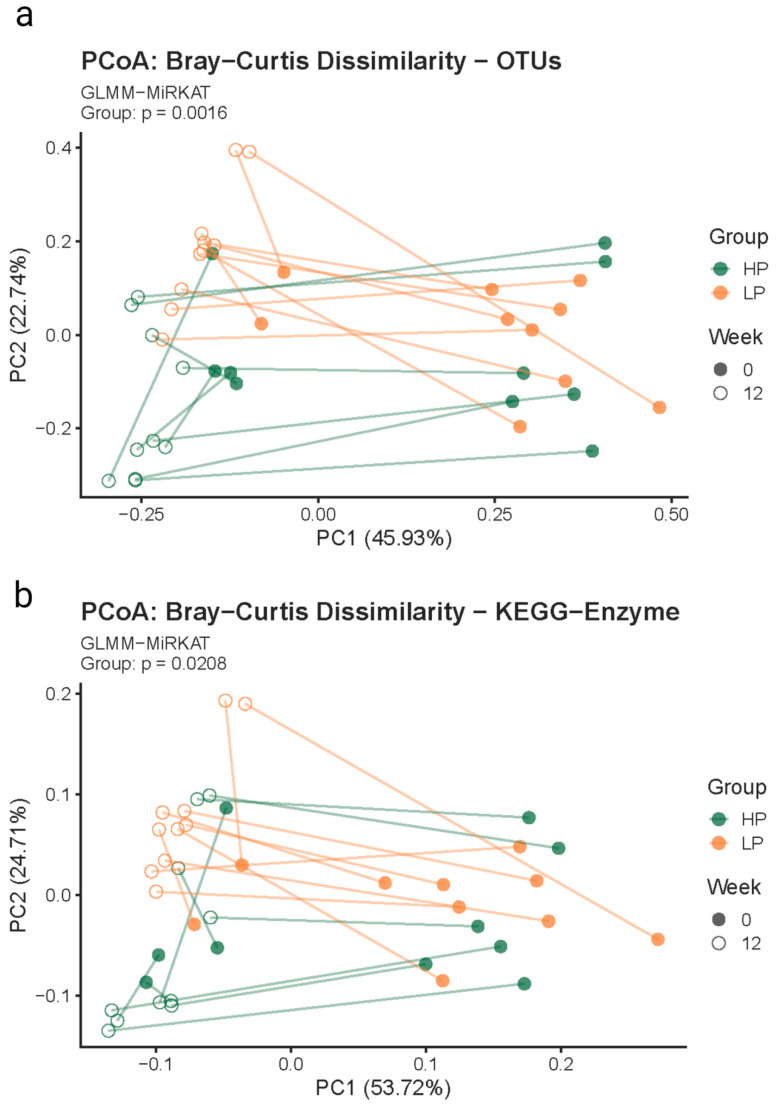
Beta diversity principal coordinates analysis plot based on Bray–Curtis dissimilarities at the OTU level (**a**) and KEGG enzyme level (**b**) for cats fed a high-protein diet (HP; green) and low-protein diet (LP; orange) at baseline (Week 0; solid circles) and 12 weeks (Week 12; open circles) after transitioning to study diet. Fecal samples corresponding to different timepoints from individual cats are connected by a line.

When accounting for both timepoints, a difference was observed between the HPD and LPD groups at both the taxonomic (*p* = 0.002) and functional levels (*p* = 0.021). Figure 2 shows the PCoA plot of the Bray–Curtis dissimilarities at the OTU and KEGG Enzyme level. At the taxonomic level, a weakly difference was observed between the HPD and LPD groups at Week 0 (*p* = 0.029) but not at Week 12 (*p* = 0.063). Conversely, at the functional level, a weakly difference was observed between the two diet groups at Week 12 (*p* = 0.035), but no difference between groups was observed at Week 0 (*p* = 0.075). No differences were observed when per-cat Week 0-to-Week 12 distances were compared across the groups (taxonomic level, *p* = 0.5; functional level, *p* = 0.9).

#### 3.2.3. Microbial Composition

**Figure 3:**
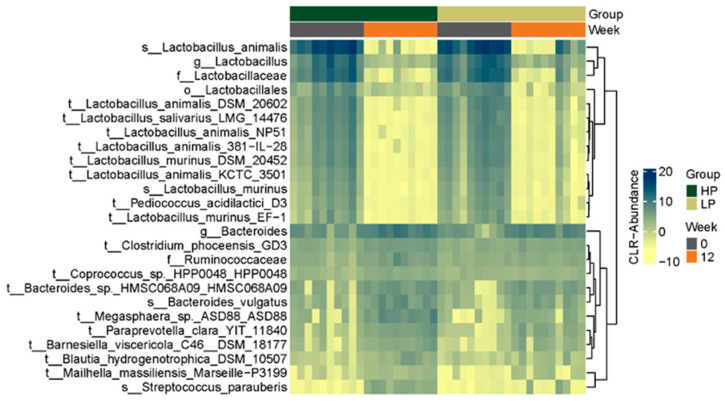
Heat map showing the top 25 differentially abundant taxa at the OTU-level in healthy cats fed either a high-protein diet (HP group, *n* = 10 cats) or a low-protein diet (LP group; *n* = 10 cats) for 12 weeks. Every fecal sample collected from each cat (10 cats/group) at Week 0 and Week 12 is represented by a column. The color gradient key displays a linear scale of abundance.

Figure 3 is a heat map that shows the top 25 differentially abundant taxa across groups at the OTU level. Heat maps representing the top 25 differentially abundant taxa at the family and genus level can be found in the Supplementary Materials (Figure S1a,b). Heat maps show similar taxonomic profiles for both groups at Week 0 prior to the transition to study diets, and a similar shift in profiles was seen after 12 weeks of feeding cats the study diets. After correcting for multiple comparisons, a significant difference in the relative abundance of several taxa was observed at all taxonomic levels (phylum, *n* = 3; family, *n* = 32; genus, *n* = 89; OTU, *n* = 483) within groups over the 12-week study period.

When comparing the fecal taxonomic composition of the LPD group with the HPD group at Week 12, the abundances of 62 OTUs were found to be significantly different between groups after correcting for multiple comparisons (Table S1). Of the OTUs, the majority belonged to the genus *Bifidobacterium* and the species *B. longum*, *B. breve.*, *B. bifidum*, and *B. kashiwanehense*, and were found to have a lower relative abundance in the HPD group compared to the LPD group.

No taxa that were differentially abundant between groups at Week 12 were associated with serum pCS concentrations. Several taxa were found to weakly correlate to either serum IS and TMAO concentrations (Figure S2); however, none of the FDR-corrected *p*-values were significant.

#### 3.2.4. KEGG Pathways

**Figure 4:**
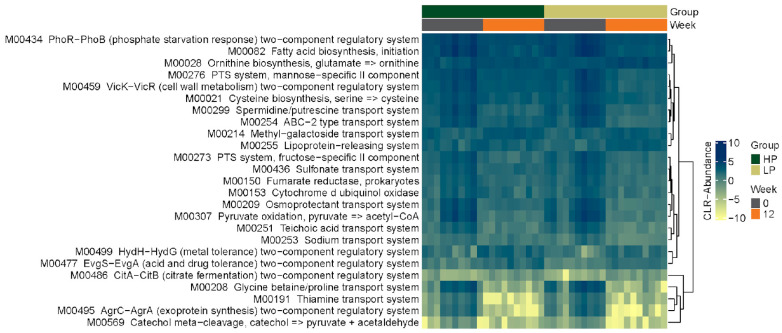
Heat map showing the top 25 differentially abundant KEGG functions in healthy cats fed either a high-protein diet (HP group; *n* = 10) or a low-protein diet (LP group; *n* = 10) for 12 weeks. Every fecal sample collected from each cat in Week 0 and Week 12 is represented by a column. The color gradient key displays a linear scale of abundance.

Figure 4 shows a heat map of the top 25 differentially abundant KEGG functions across groups at the OTU level. Of these 25 functions, the majority belong to the KEGG pathway category of ‘environmental information processing’ (18/25) followed by ‘energy metabolism’ (2/25), ‘carbohydrate and lipid metabolism’ (2/25), ‘nucleotide and amino acid metabolism’ (specifically, arginine, proline, cysteine, and methionine metabolism; 2/25), and secondary metabolism (aromatic degradation; 1/25). The shift in functional profiles over the 12-week study for both groups follows a similar pattern to the heatmaps for the taxonomic profiles (Figure 3). The functional profiles of the groups at Week 0 were similar, and a similar change in the group profiles was seen after 12 weeks of feeding cats the study diets. When functional profiles at Week 12 were compared to those at baseline within each group, several KEGG pathways were found to be significantly different for the LPD group (‘environmental information processing’, *n* = 43; ‘carbohydrate metabolism’, *n* = 18; ‘energy metabolism’, *n* = 7; ‘nucleotide and amino acid metabolism’, *n* = 11; ‘genetic information processing’, *n* = 1) and HPD group (‘environmental information processing’, *n* = 43; ‘carbohydrate metabolism’, *n* = 20; ‘energy metabolism’, *n* = 7; ‘nucleotide and amino acid metabolism’, *n* = 14; ‘genetic information processing’, *n* = 2) after correcting for multiple comparisons. When specifically looking at KEGG functions related to amino acid metabolism, cats fed the LPD had a higher relative abundance of genes for tryptophan biosynthesis (*p* = 0.029), tyrosine biosynthesis (*p* = 0.048), and phenylalanine biosynthesis (*p* = 0.027) from chorismate at Week 12 compared to Week 0. Cats fed the HPD had a higher relative abundance of genes for tyrosine biosynthesis from prephanate (*p* = 0.043) at Week 12 compared to Week 0. After multiple comparison correction, there was no difference in any KEGG function between the LPD group and HPD group at Week 12.

## 4. Discussion

In this study, we found that a change in diet and in dietary protein concentration impacts the fecal microbial composition of healthy cats. Regarding alpha diversity, we found that fecal microbial diversity within fecal samples increased in the cats fed the HPD, while no change was found in cats fed the LPD. This lack of change in alpha diversity in cats fed the LPD is attributable to a similar protein concentration between the LPD (7.4 g/100 kcal) and the pre-trial diet (8.7 g/100 kcal) whereas the HPD (11.0 g/100 kcal) has a much higher protein concentration than the pre-trial diet. At the time of enrollment, all cats were being fed the same diet, and thus, as expected, the fecal microbial diversity was not different between groups at baseline. After 12 weeks, cats fed the HPD had higher richness and diversity at both the taxonomic and functional levels than cats fed the LPD. Considering similarity in ingredients between study diets, this finding is attributable to differences in protein concentrations between the LPD and HPD.

The beta diversity analysis similarly showed a significant difference between groups at the functional level, but not the taxonomic level, at Week 12. A time-associated shift in the gut microbiome of cats within groups was observed between baseline and Week 12. This shift appears to be consistent for both groups (rather than divergent), suggesting that some microbial features shifted in similar ways. This was an expected finding associated with diet change, and is attributed to differences in ingredients, micro- and macronutrient composition, and digestibility between the baseline and study diets. Furthermore, although multiple taxa and functions were observed to be differentially abundant between all diet and timepoint combinations, most of the significant features were observed between the baseline and Week 12 fecal samples of the HPD group, thereby highlighting the marked effect of the HPD on the fecal microbiome of the cats in the present study.

We found lower fecal *Bifidobacterium* relative abundance at Week 12 in cats fed the HPD compared to those fed the LPD. This finding was consistent with current literature. *Bifidobacterium* species are considered beneficial bacteria because of their ability to promote a mucus gut barrier and ability to ferment dietary fibers in the colon into beneficial metabolites, like short-chain fatty acids [1,26]. High-protein diets favor expansion of proteolytic bacteria over saccharolytic bacteria, like bifidobacteria, and thus HPDs have repeatedly been shown to reduce fecal Bifidobacterium abundance in healthy dogs and cats [5,27,28]. Supplementing an HPD with a soluble fiber may increase bifidobacteria, as observed in dogs [29]. Studies have also shown that cats and dogs fed the HPD had greater abundance of *Clostridium* perfringens; however, this was not a finding in our study, which is likely attributable to the lower protein concentration in our HPD compared to that used in previous studies [5,27,28].

Microbial taxa that were differentially abundant between groups at Week 12 were correlated to serum uremic toxin concentrations. Although clear taxonomic differences were observed between groups at Week 12, only weak correlations were found between the differentially abundant taxa and the uremic toxins. There were no significant correlations after p-value adjustment, suggesting no clear associations between the gut microbiome and the uremic toxins in cats fed HPD versus the LPD. Fecal metagenomic analysis was only performed at baseline and Week 12, and therefore relative abundances of microbial taxa could not be correlated to serum uremic toxin concentrations at Week 4 and Week 8.

Indoxyl sulfate is a product of dietary tryptophan fermentation by the gut microbiota; this is also the case for the amino acids tyrosine and phenylalanine for pCS and choline and carnitine for TMAO. The amount of undigested protein transferred to the colon increases with the increase in dietary protein intake, and thus we hypothesized that an HPD would increase serum IS, pCS, and TMAO concentrations as a consequence of increased production by the microbiota during protein fermentation, similarly to what is observed in cats with early-stage CKD [19,30]. In IRIS stage 1 CKD cats, Jewell and Ephraim 2021 showed an increase in relative mean units of plasma IS and pCS concentrations with a diet containing 8.0 g protein per100 kcal ME or 7.0 g protein per100 kcal ME compared to a food containing 5.7 g protein per100 kcal ME [19]. Contrary to our hypothesis, we found only a mild transient increase in serum pCS concentrations after feeding healthy cats the HPD. While there appeared to be an upward trend in serum IS concentrations, particularly in the first 8 weeks, and a downward trend in serum TMAO concentrations, these findings were not statistically significant. These findings, which are subtle compared to a previous study on diet in CKD cats, may be attributable to differences in the gut microbial composition between healthy cats and those with CKD [6]. People with kidney disease have higher amounts of indole- and p-cresol-forming gut microbiota, so there may be a more profound effect with high protein intake on serum pCS and IS concentrations in cats with renal impairment versus healthy cats [8,31]. In addition, the amino acid concentrations of study diets could have impacted the potential magnitude of changes seen in serum concentrations during the study period. In non-dialyzed patients with CKD, a positive correlation was found between tyrosine and phenylalanine dietary intake and plasma pCS concentrations [32]. Oral choline supplementation in healthy people increases plasma TMAO concentrations within 1 month of supplementation [33]. Compared to the pre-trial food, the HPD had a higher concentration of tyrosine and phenylalanine, and the LPD had similar concentrations. This could be one explanation for the gradual increase in pCS concentrations during the first 8 weeks of the study in only the cats fed the HPD. The lack of a significant change in IS compared to baseline over the 12-week study in both groups of cats could be attributed to similar tryptophan concentrations between the pre-trial diet and the study diets. The concentrations of choline and carnitine in the study diets were not measured.

No adverse effects were noted for cats fed either the LPD or HPD. Serum biochemical parameters remained static throughout the 12-week study period, except for an increase in albumin and phosphorus concentrations. The increase in serum albumin concentration over the 12-week study could be due to improved protein digestibility in the study diets compared to the pre-trial diet. For cats fed the HPD, the higher protein concentration may also be a contributing factor [34]. Despite a lower dietary phosphorus concentration in the study diets (LPD: 107 mg/100 kcal; HPD: 117 mg/100 kcal) compared to the pre-trial diet (313 mg/100 kcal), the median serum phosphorus concentration increased over the 12-week study in both groups, and the increase from baseline was found to be statistically significant in the HPD group. Considering the cats exhibited normal kidney function on routine laboratory analysis and cats were fasted prior to sample collection, this finding is likely attributable to differences in diet formulation. Several factors determine absorption of phosphorus from the intestinal tract, including the source of phosphorus, relative amounts of phosphorus and calcium, and amount of 25-hydroxyvitamin D [34,35,36,37]. Inorganic sources of phosphorus (e.g., food additives) and diets with a low calcium-to-phosphorus ratio (particularly a ratio < 1:2) promote intestinal absorption of phosphorus. The study diets did not contain an inorganic phosphorus source (unlike the pre-trial diet), and the pre-trial diet had a lower calcium-to-phosphorus ratio (1:1) than the study diets (LPD: 1:7; HPD: 1.5), and therefore these factors are unlikely to contribute to the increase in serum phosphorus concentration in study cats. Differences in the digestibility of organic phosphorus sources (e.g., protein meal, bone ash) between the pre-trial diet and study diets may be a contributing factor, which was not evaluated in this study.

This study had limitations. The study lacked a control group of cats with no diet change; therefore, we cannot discern potential impacts that are due to a dietary change but may not necessarily be due to differing protein concentrations. Second, digestibility tests for pre-trial and study diets were not performed in this study. The impact of dietary protein on the gut microbiome and microbial metabolites likely depends on protein concentration, source, and digestibility. For example, foods with poorly digestible protein increase the amount of dietary protein available for putrefaction by microbial populations in the colon [30]. Third, cats were group-housed and fed ad libitum during the study. It was not feasible to feed them based on calculated energy requirements in the housing facility as this would have put undue stress on the cats. Therefore, total protein dietary intake could have varied between cats within the same group. Lastly, the biological variation in serum IS, pCS, and TMAO has been previously described in cats [20]. The within-individual variation in serum concentrations was found to be high for these toxins, especially IS and pCS. Therefore, the gradual increase in pCS concentration after feeding cats an HPD could be attributed to normal intraindividual variation rather than a significant biological change.

## 5. Conclusions

The results of this study suggest that both diet change and dietary protein concentration shift the fecal microbial community and microbial functions in healthy adult cats. Cats fed the HPD had increased diversity of the fecal microbial community and reduced relative abundance of beneficial bacteria in the *Bifidobacterium* genus. Additionally, the HPD increased serum concentrations of the uremic toxin pCS; however, the effect was short-lived. Further study is needed to determine the impact of these findings on the health of cats with disease.

## Figures and Tables

**Table 1:** Food composition of the pre-trial commercial diet, low-protein study diet, and high-protein study diet.

Nutrient		Pre-Trial Diet ^1^	Low-Protein Diet ^2^	High-Protein Diet ^3^
	Caloric Density, Macronutrient, and Mineral Content			
Caloric density DM	kcal/100 gr	389	415	413
Protein	g/100 kcal	8.7	7.4	11.0
Fat	g/100 kcal	3.6	4.6	4.4
Carbohydrate	g/100 kcal	11.1	10.1	6.8
Soluble fiber	g/100 kcal	0.4	0.4	0.3
Insoluble fiber	g/100 kcal	2.4	2.2	2.4
Total fiber	g/100 kcal	2.8	2.7	2.7
Calcium	mg/100 kcal	348.5	185.2	179.5
Phosphorus	mg/100 kcal	313.2	106.8	117.0
Potassium	mg/100 kcal	209.5	340.8	378.6
Sodium	mg/100 kcal	117.6	74.2	72.2
Magnesium	mg/100 kcal	36.6	23.8	23.4
	Amino Acid Concentration			
Alanine	g/100 kcal	0.56	0.43	0.72
Arginine	g/100 kcal	0.49	0.37	0.49
Aspartic acid	g/100 kcal	0.70	0.63	0.86
Cysteine	g/100 kcal	0.13	0.11	0.17
Glutamic acid	g/100 kcal	1.59	1.21	2.04
Glycine	g/100 kcal	0.50	0.30	0.40
Histidine	g/100 kcal	0.20	0.18	0.26
Isoleucine	g/100 kcal	0.33	0.30	0.45
Leucine	g/100 kcal	0.81	0.68	1.23
Lysine	g/100 kcal	0.43	0.44	0.59
Methionine	g/100 kcal	0.17	0.17	0.26
Phenylalanine	g/100 kcal	0.42	0.37	0.60
Proline	g/100 kcal	0.57	0.41	0.73
Serine	g/100 kcal	0.39	0.34	0.52
Threonine	g/100 kcal	0.31	0.29	0.41
Tyrosine	g/100 kcal	0.30	0.27	0.47
Tryptophan	g/100 kcal	0.06	0.07	0.08
Valine	g/100 kcal	0.43	0.39	0.57

^1^ Ingredient list for pre-trial food in order of preponderance is as follows: ground yellow corn, corn gluten meal, chicken by-product meal, soybean meal, beef tallow preserved with mixed tocopherols, animal digest, calcium carbonate, turkey by-product meal, salmon meal, ocean fish meal, phosphoric acid, choline chloride, salt, titanium dioxide, potassium chloride, vitamin E supplement, niacin supplement, vitamin A supplement, d-calcium pantothenate, thiamine mononitrate (vitamin B-2), pyridoxine hydrochloride (vitamin B-6), menadione sodium bisulfite complex (vitamin K), vitamin D3 supplement, folic acid, biotin, vitamin B-12 supplement, ferrous sulfate, zinc oxide, manganous oxide, copper sulfate, calcium iodate, sodium selenite, taurine, yellow 6, yellow 5, red 40, blue 2, rosemary extract. ^2^ Ingredient list for LPD in order of preponderance is as follows: brewers rice, corn, tuna meat, soybean meal, beef tallow preserved with vitamin E, corn gluten meal, potato protein, dried eggs, yeast, animal digest, cellulose, fish oil, calcium carbonate, potassium chloride, potassium citrate monohydrate, L-lysine, choline chloride, salt, taurine, DL-methionine, vitamin E, zinc sulfate, ferrous sulfate, thiamine, mononitrate (vitamin B-1), niacin (vitamin B-3), manganese sulfate, vitamin A supplement, calcium pantothenate (vitamin B-5), copper sulfate, riboflavin supplement (vitamin B-2), vitamin B-12 supplement, pyridoxine hydrochloride (vitamin B-6), folic acid (vitamin B-9), vitamin D-3 supplement, calcium iodate, biotin (vitamin B-7), menadione sodium bisulfite complex (vitamin K), sodium selenite. ^3^ Ingredient list for HPD in order of preponderance is as follows: corn gluten meal, soybean meal, brewers rice, tuna meat, beef tallow preserved with vitamin E, potato protein, corn, dried eggs, yeast, animal digest, cellulose, fish oil, calcium carbonate, potassium chloride, L-lysine, potassium citrate monohydrate, choline chloride, salt, taurine, DL-methionine, vitamin E, zinc sulfate, ferrous sulfate, thiamine mononitrate (vitamin B-1), niacin (vitamin B-3), manganese sulfate, vitamin A supplement, calcium pantothenate (vitamin B-5), copper sulfate, riboflavin supplement (vitamin B-2), vitamin B-12 supplement, pyridoxine hydrochloride (vitamin B-6), folic acid (vitamin B-9), vitamin D-3 supplement, calcium iodate, biotin (vitamin B-7), menadione sodium bisulfite complex (vitamin K), sodium selenite.

**Table 2:** Summary of physical examination parameters (body weight, BCS, MCS), fecal score, select serum biochemistry values at baseline (Week 0) and after 3 months of eating a low-protein diet or high-protein diet (Week 12). Data presented as median and range.

		Low-Protein Diet (*n* = 10)		High-Protein Diet (*n* = 10)	
	Reference Interval	Week 0	Week 12	Week 0	Week 12
Body weight (kg)		5.0 (2.9–6.7)	4.9 (3.0–6.6)	4.5 (3.0–6.1)	4.4 (2.8–6.0)
BCS	0–9	6 (5–8)	6 (5–8)	5.5 (5–6)	6 (5–7)
MCS	0–3	0.5 (0–1)	1 (0–1)	0 (0–1)	0 (0–1)
Fecal score	1–7	2.5 (2–3)	2 (2–4)	3 (2–4)	2 (2–3)
Blood urea nitrogen	18–35 mg/dL	25 (21–31)	26 (20–28)	25 (21–34)	24 (18–32)
Creatinine	0.8–2.4 mg/dL	1.4 (1.0–1.7)	1.4 (1.1–2.0)	1.4 (1.0–1.7)	1.4 (1.0–1.8)
Albumin	3.1–4.4 g/dL	3.5 (3.2–3.7) ^a^	3.8 (3.2–4.0) ^b^	3.3 (3.2–4.2) ^a^	3.7 (3.2–4.7) ^b^
Phosphorus	3.0–6.0 mg/dL	4.1 (3.5–5.2)	5.2 (3.0–6.1)	3.9 (3.3–4.4) ^a^	5.1 (4.3–6.3) ^b^
Calcium	9.2–11.1 mg/dL	9.7 (9.1–10.1)	9.7 (8.6–10.9)	9.6 (9.3–10.0)	9.7 (9.4–11.0)
Urine specific gravity	>1.035	1.056 (1.032–1.066)	1.053 (1.044–1.057)	1.055 (1.050–1.066)	1.052 (1.044–1.068)

For each row, columns within groups (low-protein diet and high-protein diet) bearing a different superscript letter were significantly different from each other (*p* < 0.05).

**Table 3:** Serum p-cresol sulfate, indoxyl sulfate, and trimethylamine-n-oxide concentrations at baseline (Week 0) and after 4, 8, and 12 weeks of eating a low-protein diet or high-protein diet. Data presented as median and range.

	Low-Protein Diet (*n* = 10)	High-Protein Diet (*n* = 10)
p-Cresol Sulfate		
Week 0	26,288 (7391–51,217)	21,184 (13,171–66,635) *
Week 4	32,647 (16,506–57,342)	35,851 (9944–86,416)
Week 8	32,944 (14,727–52,337)	41,969 (14,438–83,490) *
Week 12	27,942 (14,419–46,022)	27,449 (8158–49,244)
Indoxyl Sulfate		
Week 0	817 (428–2945)	1147 (736–4755)
Week 4	964 (500–2216)	1802 (942–3079)
Week 8	1167 (688–1710)	1537 (1201–2982)
Week 12	1053 (702–2336)	1449 (796–2429)
Trimethylamine-n-oxide		
Week 0	1660 (932–2703)	1932 (1326–3867)
Week 4	1602 (862–3155)	1631 (1086–2665)
Week 8	1523 (1035–2761)	1593 (1338–2531)
Week 12	1357 (915–1614)	1741 (684–2322)

* Asterisk signifies a significant difference was found between serum concentrations (*p* < 0.05).

**Table 4:** Observed operational taxonomic unit (OTU) richness, Chao1, and Shannon diversity index at the taxonomic and KEGG functional level at baseline (Week 0) and after 12 weeks (Week 12) of eating a low-protein diet or high-protein diet. Data presented as median and range.

	Low-Protein Diet (*n* = 10)		High-Protein Diet (*n* = 10)	
	Week 0	Week 12	Week 0	Week 12
	Taxonomic Level			
Observed OTU	633 (482–735)	618 (486–723)	639 (385–720)	662 (385–710)
Chao1	702 (524–790)	664 (562–796)	719 (461–774)	695 (621–732)
Shannon	2.9 (2.2–3.8)	3.3 (2.7–3.5)	3.2 (2.6–3.7) ^a^	3.5 (3.3–4.1) ^b^
	KEGG Functional Level			
Observed OTUs	1431 (1217–1644)	1440 (1310–1569)	1452 (1116–1548) ^a^	1532 (1470–1569) ^b^
Chao1	1544 (1311–1780)	1526 (1397–1646)	1537 (1346–1657)	1605 (1470–1653)
Shannon	6.5 (6.2–6.6)	6.5 (6.4–6.6)	6.5 (6.3–6.6) ^a^	6.6 (6.5–6.7) ^b^

For each row, columns within groups (low-protein diet and high-protein diet) bearing a different superscript letter were significantly different from each other (*p* < 0.05).

## Data Availability

The sequences were deposited in the National Institutes of Health Sequence Read Archive under accession number PRJNA786887.

## Supplementary Materials

The following supporting information can be downloaded at: https://www.mdpi.com/article/10.3390/vetsci10080497/s1. Figure S1: Heat map representing the top 25 significant taxa at the genus level (a) and family level (b) for healthy cats fed either a high-protein diet (HP group, *n* = 10 cats) or a low-protein diet (LP group; *n* = 10 cats) for 12 weeks. Every fecal sample collected from each cat at Week 0 and Week 12 is represented by a column. The color gradient key displays a linear scale of abundance; Figure S2: Correlation matrix between Week 12 differential taxa and uremic toxins. The correlation analysis was focused on taxa that were differentially abundant between the low-protein diet and high-protein diet groups at Week 12. Legend represents the correlation coefficient for significant correlations (*p* < 0.05; p-value not adjusted for multiple comparisons; Table S1: List of taxa at the OTU level that were significantly (*p* < 0.05) different after 12 weeks of feeding either a high-protein diet or low-protein diet in healthy cats.
