# Supplementary material for: The Effect of Dietary Protein Concentration on the Fecal Microbiome and Serum Concentrations of Gut-Derived Uremic Toxins in Healthy Adult Cats
Source: Vet Sci. 2023 Aug 2;10(8):497. doi: 10.3390/vetsci10080497 (PMC10457753; doi:10.3390/vetsci10080497)
Supplement: Supplementary file 1 [file vetsci-10-00497-s001.zip › Table S1.pdf]

Table S1. List of taxa at the OTU level that were significantly (P<0.05) different between healthy cats fed either a high protein diet or low protein diet for 12 weeks.

Taxon	Adjusted P-value
k__Bacteria;p__Actinobacteria;c__Actinobacteria;o__Bifidobacteriales;f__Bifidobacteriaceae;g__Bifidobacterium;s__Bifidobacterium_longum;t__Bifidobacterium_longum_JCM_1217	0.007038
k__Bacteria;p__Actinobacteria;c__Actinobacteria;o__Bifidobacteriales;f__Bifidobacteriaceae;g__Bifidobacterium;s__Bifidobacterium_longum;t__Bifidobacterium_longum_BT1	0.007038
k__Bacteria;p__Actinobacteria;c__Actinobacteria;o__Bifidobacteriales;f__Bifidobacteriaceae;g__Bifidobacterium;s__Bifidobacterium_longum;t__Other	0.007061
k__Bacteria;p__Actinobacteria;c__Actinobacteria;o__Bifidobacteriales;f__Bifidobacteriaceae;g__Bifidobacterium;s__Bifidobacterium_longum;t__Bifidobacterium_longum_35624	0.00707
k__Bacteria;p__Actinobacteria;c__Actinobacteria;o__Bifidobacteriales;f__Bifidobacteriaceae;g__Bifidobacterium;s__Bifidobacterium_longum;t__Bifidobacterium_longum_CECT_7210	0.007165
k__Bacteria;p__Actinobacteria;c__Actinobacteria;o__Bifidobacteriales;f__Bifidobacteriaceae;g__Bifidobacterium;s__Bifidobacterium_reuteri;t__Bifidobacterium_reuteri_DSM_23975	0.007184
k__Bacteria;p__Actinobacteria;c__Actinobacteria;o__Bifidobacteriales;f__Bifidobacteriaceae;g__Bifidobacterium;s__Bifidobacterium_longum;t__Bifidobacterium_longum_NCIMB8809	0.007229
k__Bacteria;p__Actinobacteria;c__Actinobacteria;o__Bifidobacteriales;f__Bifidobacteriaceae;g__Bifidobacterium;s__Bifidobacterium_longum;t__Bifidobacterium_longum_NCC2705	0.007248
k__Bacteria;p__Actinobacteria;c__Actinobacteria;o__Bifidobacteriales;f__Bifidobacteriaceae;g__Bifidobacterium;s__Bifidobacterium_breve;t__Other	0.00733
k__Bacteria;p__Actinobacteria;c__Actinobacteria;o__Bifidobacteriales;f__Bifidobacteriaceae;g__Bifidobacterium;s__Bifidobacterium_longum;t__Bifidobacterium_longum_KACC_91563	0.007777
k__Bacteria;p__Actinobacteria;c__Actinobacteria;o__Bifidobacteriales;f__Bifidobacteriaceae;g__Bifidobacterium;s__Bifidobacterium_longum;t__Bifidobacterium_longum_CCUG30698	0.008325
k__Bacteria;p__Actinobacteria;c__Actinobacteria;o__Bifidobacteriales;f__Bifidobacteriaceae;g__Bifidobacterium;s__Bifidobacterium_longum;t__Bifidobacterium_longum_DJO10A	0.008809
k__Bacteria;p__Actinobacteria;c__Actinobacteria;o__Bifidobacteriales;f__Bifidobacteriaceae;g__Bifidobacterium;s__Bifidobacterium_longum;t__Bifidobacterium_longum_GT15	0.008943
k__Bacteria;p__Actinobacteria;c__Actinobacteria;o__Bifidobacteriales;f__Bifidobacteriaceae;g__Bifidobacterium;s__Bifidobacterium_breve;t__Bifidobacterium_breve_NRBB57	0.008949

k__Bacteria;p__Actinobacteria;c__Actinobacteria;o__Bifidobacteriales;f__Bifidobacteriaceae;g__Bifidobacterium;s__Bifidobacterium_breve;t__Bifidobacterium_breve_DRBB26	0.009604
k__Bacteria;p__Actinobacteria;c__Actinobacteria;o__Bifidobacteriales;f__Bifidobacteriaceae;g__Bifidobacterium;s__Bifidobacterium_breve;t__Bifidobacterium_breve_NRBB09	0.009921
k__Bacteria;p__Actinobacteria;c__Actinobacteria;o__Bifidobacteriales;f__Bifidobacteriaceae;g__Bifidobacterium;s__Bifidobacterium_sp._12_1_47BFAA;t__Bifidobacterium_sp._12_1_47BFAA_12_1_47BFAA	0.010235
k__Bacteria;p__Actinobacteria;c__Actinobacteria;o__Bifidobacteriales;f__Bifidobacteriaceae;g__Bifidobacterium;s__Bifidobacterium_breve;t__Bifidobacterium_breve_JCM_7019	0.011109
k__Bacteria;p__Actinobacteria;c__Actinobacteria;o__Bifidobacteriales;f__Bifidobacteriaceae;g__Bifidobacterium;s__Bifidobacterium_breve;t__Bifidobacterium_breve_12L	0.011278
k__Bacteria;p__Actinobacteria;c__Actinobacteria;o__Bifidobacteriales;f__Bifidobacteriaceae;g__Bifidobacterium;s__Bifidobacterium_saguini;t__Bifidobacterium_saguini_DSM_23967	0.011325
k__Bacteria;p__Actinobacteria;c__Actinobacteria;o__Bifidobacteriales;f__Bifidobacteriaceae;g__Bifidobacterium;s__Bifidobacterium_bifidum;t__Other	0.013131
k__Bacteria;p__Actinobacteria;c__Actinobacteria;o__Bifidobacteriales;f__Bifidobacteriaceae;g__Bifidobacterium;s__Bifidobacterium_breve;t__Bifidobacterium_breve_S27	0.01343
k__Bacteria;p__Actinobacteria;c__Actinobacteria;o__Bifidobacteriales;f__Bifidobacteriaceae;g__Bifidobacterium;s__Bifidobacterium_longum;t__Bifidobacterium_longum_BG7	0.013471
k__Bacteria;p__Actinobacteria;c__Actinobacteria;o__Bifidobacteriales;f__Bifidobacteriaceae;g__Bifidobacterium;s__Bifidobacterium_breve;t__Bifidobacterium_breve_NRBB02	0.014306
k__Bacteria;p__Actinobacteria;c__Actinobacteria;o__Bifidobacteriales;f__Bifidobacteriaceae;g__Bifidobacterium;s__Other;t__Other	0.014364
k__Bacteria;p__Actinobacteria;c__Actinobacteria;o__Bifidobacteriales;f__Bifidobacteriaceae;g__Bifidobacterium;s__Bifidobacterium_thermophilum;t__Bifidobacterium_thermophilum_RBL67	0.01592
k__Bacteria;p__Actinobacteria;c__Actinobacteria;o__Bifidobacteriales;f__Bifidobacteriaceae;g__Bifidobacterium;s__Bifidobacterium_longum;t__Bifidobacterium_longum_F8	0.016403
k__Bacteria;p__Actinobacteria;c__Actinobacteria;o__Bifidobacteriales;f__Bifidobacteriaceae;g__Bifidobacterium;s__Bifidobacterium_breve;t__Bifidobacterium_breve_LMC520	0.016663
k__Bacteria;p__Actinobacteria;c__Actinobacteria;o__Bifidobacteriales;f__Bifidobacteriaceae;g__Bifidobacterium;s__Bifidobacterium_longum;t__Bifidobacterium_longum_105-A	0.016826
k__Bacteria;p__Actinobacteria;c__Actinobacteria;o__Bifidobacteriales;f__Bifidobacteriaceae;g__Bifidobacterium;s__Bifidobacterium_breve;t__Bifidobacterium_breve_NRBB11	0.017975
k__Bacteria;p__Actinobacteria;c__Actinobacteria;o__Bifidobacteriales;f__Bifidobacteriaceae;g__Bifidobacterium;s__Bifidobacterium_longum;t__Bifidobacterium_longum_157F	0.017996

k__Bacteria;p__Firmicutes;c__Other;o__Other;f__Other;g__Other;s__Other;t__Other	0.018374
k__Bacteria;p__Actinobacteria;c__Actinobacteria;o__Bifidobacteriales;f__Bifidobacteriaceae;g__Bifidobacterium;s__Bifidobacterium_longum;t__Bifidobacterium_longum_BXY01	0.018808
k__Bacteria;p__Actinobacteria;c__Actinobacteria;o__Bifidobacteriales;f__Bifidobacteriaceae;g__Bifidobacterium;s__Bifidobacterium_kashiwanohense;t__Other	0.019349
k__Bacteria;p__Actinobacteria;c__Actinobacteria;o__Bifidobacteriales;f__Bifidobacteriaceae;g__Bifidobacterium;s__Bifidobacterium_bifidum;t__Bifidobacterium_bifidum_NCIMB_41171	0.019706
k__Bacteria;p__Actinobacteria;c__Actinobacteria;o__Bifidobacteriales;f__Bifidobacteriaceae;g__Bifidobacterium;s__Bifidobacterium_breve;t__Bifidobacterium_breve_UCC2003	0.021219
k__Bacteria;p__Actinobacteria;c__Actinobacteria;o__Bifidobacteriales;f__Bifidobacteriaceae;g__Bifidobacterium;s__Bifidobacterium_kashiwanohense;t__Bifidobacterium_kashiwanohense_PV20-2	0.021782
k__Bacteria;p__Actinobacteria;c__Actinobacteria;o__Bifidobacteriales;f__Bifidobacteriaceae;g__Bifidobacterium;s__Bifidobacterium_kashiwanohense;t__Bifidobacterium_kashiwanohense_JCM_15439	0.022786
k__Bacteria;p__Actinobacteria;c__Actinobacteria;o__Bifidobacteriales;f__Bifidobacteriaceae;g__Bifidobacterium;s__Bifidobacterium_bifidum;t__Bifidobacterium_bifidum_PRI_1	0.025354
k__Bacteria;p__Firmicutes;c__Clostridia;o__Clostridiales;f__Ruminococcaceae;g__Faecalibacterium;s__Faecalibacterium_prausnitzii;t__Faecalibacterium_prausnitzii_2789STDY5834970	0.026432
k__Bacteria;p__Firmicutes;c__Erysipelotrichia;o__Erysipelotrichales;f__Erysipelotrichaceae;g__Faecalitalea;s__Faecalitalea_cylindroides;t__Faecalitalea_cylindroides_T2-87	0.029521
k__Bacteria;p__Actinobacteria;c__Actinobacteria;o__Bifidobacteriales;f__Bifidobacteriaceae;g__Bifidobacterium;s__Bifidobacterium_longum;t__Bifidobacterium_longum_AH1206	0.029735
k__Bacteria;p__Actinobacteria;c__Actinobacteria;o__Bifidobacteriales;f__Bifidobacteriaceae;g__Bifidobacterium;s__Bifidobacterium_bifidum;t__Bifidobacterium_bifidum_2789STDY5608877	0.030296
k__Bacteria;p__Firmicutes;c__Negativicutes;o__Veillonellales;f__Veillonellaceae;g__Megasphaera;s__Megasphaera_elsdenii;t__Megasphaera_elsdenii_T81	0.030789
k__Bacteria;p__Actinobacteria;c__Actinobacteria;o__Bifidobacteriales;f__Bifidobacteriaceae;g__Bifidobacterium;s__Bifidobacterium_bifidum;t__Bifidobacterium_bifidum_BGN4	0.032044
k__Bacteria;p__Firmicutes;c__Clostridia;o__Clostridiales;f__Ruminococcaceae;g__Faecalibacterium;s__Faecalibacterium_prausnitzii;t__Other	0.033175
k__Bacteria;p__Actinobacteria;c__Actinobacteria;o__Bifidobacteriales;f__Bifidobacteriaceae;g__Bifidobacterium;s__Bifidobacterium_breve;t__Bifidobacterium_breve_689b	0.034435
k__Bacteria;p__Firmicutes;c__Clostridia;o__Clostridiales;f__Other;g__Intestinimonas;s__Intestinimonas_massiliensis;t__Intestinimonas_massiliensis_GD2	0.039188

k__Bacteria;p__Firmicutes;c__Clostridia;o__Clostridiales;f__Ruminococcaceae;g__Agathobaculum;s__Agathobaculum_desmolans;t__Agathobaculum_desmolans_ATCC_43058	0.040182
k__Bacteria;p__Actinobacteria;c__Actinobacteria;o__Bifidobacteriales;f__Bifidobacteriaceae;g__Bifidobacterium;s__Bifidobacterium_adolescentis;t__Bifidobacterium_adolescentis_2789STDY5834850	0.040415
k__Bacteria;p__Actinobacteria;c__Actinobacteria;o__Bifidobacteriales;f__Bifidobacteriaceae;g__Bifidobacterium;s__Bifidobacterium_bifidum;t__Bifidobacterium_bifidum_BF3	0.040705
k__Bacteria;p__Actinobacteria;c__Actinobacteria;o__Bifidobacteriales;f__Bifidobacteriaceae;g__Bifidobacterium;s__Bifidobacterium_breve;t__Bifidobacterium_breve_JCM_7017	0.04097
k__Bacteria;p__Firmicutes;c__Clostridia;o__Clostridiales;f__Lachnospiraceae;g__Roseburia;s__Roseburia_inulinivorans;t__Other	0.042152
k__Bacteria;p__Firmicutes;c__Clostridia;o__Clostridiales;f__Lachnospiraceae;g__Blautia;s__Blautia_sp._Marseille-P3087;t__Blautia_sp._Marseille-P3087_Marseille-P3087	0.043359
k__Bacteria;p__Actinobacteria;c__Actinobacteria;o__Bifidobacteriales;f__Bifidobacteriaceae;g__Bifidobacterium;s__Bifidobacterium_adolescentis;t__Bifidobacterium_adolescentis_ATCC_15703	0.043801
k__Bacteria;p__Firmicutes;c__Clostridia;o__Clostridiales;f__Ruminococcaceae;g__Ruminiclostridium;s__[Clostridium]_leptum;t__[Clostridium]_leptum_DSM_753	0.04411
k__Bacteria;p__Firmicutes;c__Negativicutes;o__Acidaminococcales;f__Acidaminococcaceae;g__Phascolarctobacterium;s__Phascolarctobacterium_succinatutens;t__Phascolarctobacterium_succinatutens_YIT_12067	0.044675
k__Bacteria;p__Firmicutes;c__Clostridia;o__Clostridiales;f__Eubacteriaceae;g__Eubacterium;s__Eubacterium_sp._3_1_31;t__Eubacterium_sp._3_1_31_3_1_31	0.045296
k__Bacteria;p__Actinobacteria;c__Actinobacteria;o__Bifidobacteriales;f__Bifidobacteriaceae;g__Bifidobacterium;s__Bifidobacterium_breve;t__Bifidobacterium_breve_NCFB_2258	0.046181
k__Bacteria;p__Firmicutes;c__Clostridia;o__Clostridiales;f__Lachnospiraceae;g__Lachnoclostridium;s__Other;t__Other	0.046353
k__Bacteria;p__Firmicutes;c__Erysipelotrichia;o__Erysipelotrichales;f__Erysipelotrichaceae;g__Merdibacter;s__Merdibacter_massiliensis;t__Merdibacter_massiliensis_Marseille-P3254	0.047958
k__Bacteria;p__Firmicutes;c__Clostridia;o__Clostridiales;f__Ruminococcaceae;g__Gemmiger;s__Gemmiger_formicilis;t__Gemmiger_formicilis_ATCC_27749	0.048325
k__Bacteria;p__Actinobacteria;c__Actinobacteria;o__Bifidobacteriales;f__Bifidobacteriaceae;g__Bifidobacterium;s__Bifidobacterium_sp._TRE_D;t__Bifidobacterium_sp._TRE_D_TRE_D	0.048713
