# Supplementary figures and images for: The Effect of Dietary Protein Concentration on the Fecal Microbiome and Serum Concentrations of Gut-Derived Uremic Toxins in Healthy Adult Cats
Source: Vet Sci. 2023 Aug 2;10(8):497. doi: 10.3390/vetsci10080497 (PMC10457753; doi:10.3390/vetsci10080497)

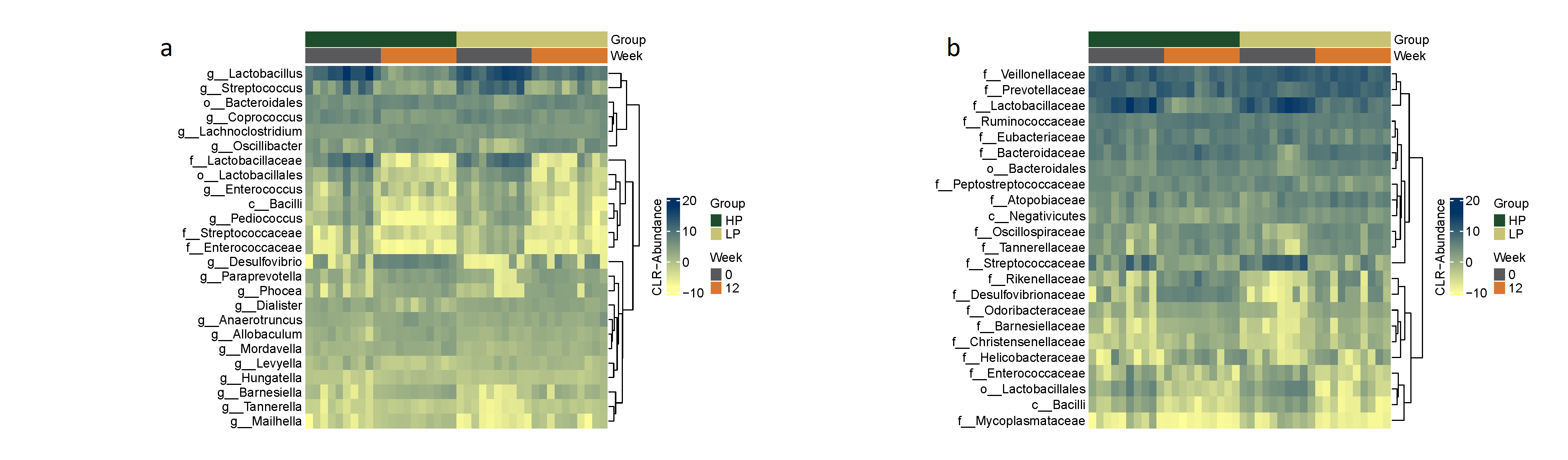

Supplement: Supplementary file 1 [file vetsci-10-00497-s001.zip › Figure S1.png]

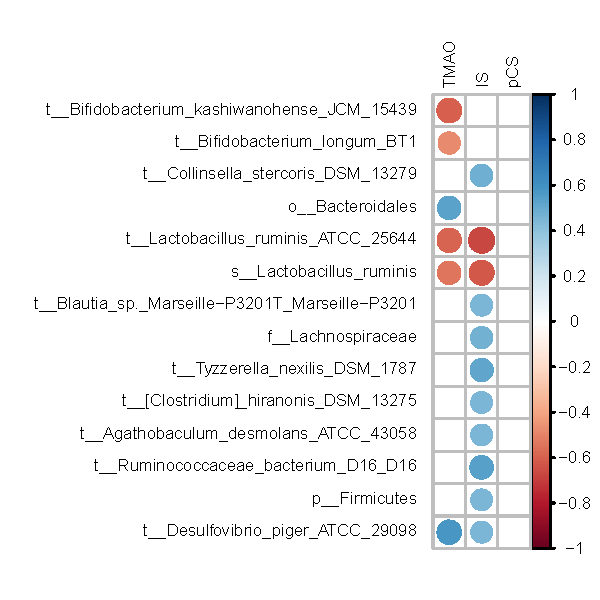

Supplement: Supplementary file 1 [file vetsci-10-00497-s001.zip › Figure S2.png]
